# Sex Differences in Photoprotective Responses to 1,25-Dihydroxyvitamin D3 in Mice Are Modulated by the Estrogen Receptor-β

**DOI:** 10.3390/ijms22041962

**Published:** 2021-02-16

**Authors:** Wannit Tongkao-on, Chen Yang, Bianca Y. McCarthy, Warusavithana G. Manori De Silva, Mark S. Rybchyn, Clare Gordon-Thomson, Katie M. Dixon, Gary M. Halliday, Vivienne E. Reeve, Rebecca S. Mason

**Affiliations:** 1 Department of Physiology, University of Sydney, Sydney, NSW 2006, Australia; may.star.edu@gmail.com (W.T.-o.); chenyangashley@gmail.com (C.Y.); Bianca.McCarthy@Lubrizol.com (B.Y.M.); mano1202au@yahoo.com (W.G.M.D.S.); m.rybchyn@unsw.edu.au (M.S.R.); clare.gordon-thomson@sydney.edu.au (C.G.-T.); 2 Anatomy and Histology, University of Sydney, Sydney, NSW 2006, Australia; katie.dixon@sydney.edu.au; 3 Dermatology, Faculty of Medicine and Health, University of Sydney, Sydney, NSW 2006, Australia; gary.halliday1@bigpond.com; 4 Faculty of Veterinary Science, University of Sydney, Sydney, NSW 2006, Australia; vivienne.reeve@sydney.edu.au

**Keywords:** 1α,25-dihydroxyvitaminD3, photoprotection, DNA damage, cyclobutane pyrimidine dimers, edema, photoimmune suppression, female vs. male mice, ER-β knockout

## Abstract

Susceptibility to photoimmune suppression and photocarcinogenesis is greater in male than in female humans and mice and is exacerbated in female estrogen receptor-beta knockout (ER-β−/−) mice. We previously reported that the active vitamin D hormone, 1,25-dihydroxyvitamin D3 (1,25(OH)2D), applied topically protects against the ultraviolet radiation (UV) induction of cutaneous cyclobutane pyrimidine dimers (CPDs) and the suppression of contact hypersensitivity (CHS) in female mice. Here, we compare these responses in female versus male Skh:hr1 mice, in ER-β−/−/−− versus wild-type C57BL/6 mice, and in female ER-blockaded Skh:hr1 mice. The induction of CPDs was significantly greater in male than female Skh:hr1 mice and was more effectively reduced by 1,25(OH)2D in female Skh:hr1 and C57BL/6 mice than in male Skh:hr1 or ER-β−/− mice, respectively. This correlated with the reduced sunburn inflammation due to 1,25(OH)2D in female but not male Skh:hr1 mice. Furthermore, although 1,25(OH)2D alone dose-dependently suppressed basal CHS responses in male Skh:hr1 and ER-β−/− mice, UV-induced immunosuppression was universally observed. In female Skh:hr1 and C57BL/6 mice, the immunosuppression was decreased by 1,25(OH)2D dose-dependently, but not in male Skh:hr1, ER-β−/−, or ER-blockaded mice. These results reveal a sex bias in genetic, inflammatory, and immune photoprotection by 1,25(OH)2D favoring female mice that is dependent on the presence of ER-β.

## 1. Introduction

Although vitamin D is best known for its actions in increasing gut calcium absorption to facilitate bone and muscle function [1], there are vitamin D receptors (VDR) in virtually all nucleated cells [2], and vitamin D metabolites have effects in many other systems, including cardiovascular effects on blood pressure and endothelial function [3]; effects on the immune system, including anti-inflammatory effects [4]; and some anti-cancer actions [1]. There is some evidence to suggest that the activation of the vitamin D system in skin plays a protective role in skin cancers [5,6].

The major risk factors for skin carcinogenesis induced by ultraviolet radiation (UV) exposure are unrepaired pre-mutagenic DNA damage, characterized primarily by the cyclobutane pyrimidine dimer (CPD); the upregulation of reactive oxygen and nitrogen species; and the release of anti-inflammatory cytokines that, together with DNA damage, mediate immunosuppression [7,8,9,10,11]. Experimental evidence suggests that 1,25-dihydroxyvitamin D3 (1,25(OH)2D), which is synthesized in skin after sunlight exposure [12,13], acts physiologically to protect skin from UV photodamage [14,15]. We and others have shown that solar-simulated UV (SSUV)-induced DNA damage, including CPDs, was reduced after topical treatment with 1,25(OH)2D or related analogs in Skh:hr1 mice and humans [6,16,17,18,19,20,21,22]. Furthermore, the severity of both the suppression of contact hypersensitivity (CHS) and of photocarcinogenesis induced in mice by SSUV was reduced by topical vitamin D compounds [6,23,24]. These observations are consistent with the reported inhibition of chemical carcinogenesis by vitamin D [25,26] and the exacerbation of photocarcinogenesis in vitamin D receptor (VDR) knockout mice [27].

Both the VDR and estrogen receptor-β (ER-β) are expressed in skin, and there is evidence that both contribute to photoprotection [27,28]. Increased male susceptibility for UV-induced immunosuppression has been reported in both mice [29] and humans [30], and for photocarcinogenesis in mice [31], findings that are consistent with the greater incidence and mortality reported for skin cancer in men [32,33]. Topical treatment with 17β-estradiol or the phytoestrogenic isoflavone equol protected human subjects [34] and mice against photoimmune suppression, reversed in mice by the ER antagonist ICI 182,780 [35,36], and against photocarcinogenesis [37], indicating an ER signaling involvement. The non-classical ER-β was implicated by the exacerbated photoimmune suppression and enhanced tumor growth observed in ER-β−/− mice compared with wild-type mice [38,39].

As ER-β signaling upregulates the expression of the VDR [40], it is likely that there is some interaction between the photoprotective pathways of 1,25(OH)2D and ER-β. In our previous studies, the protective effect of 1,25(OH)2D against photoimmune suppression was demonstrated in female Skh:hr1 mice but was not examined in males because of the likelihood of fighting, which could otherwise induce skin damage artifacts. Here we propose that there is a sex-related modulation of the 1,25(OH)2D protective effect. We tested this in female and male Skh:hr1 mice, which lack ER-α receptors in the skin [36,38] and examined a potential mechanism by also studying protection from UV-induced DNA damage and immune suppression by 1,25(OH)2D in two further models: in female ER-β knockout and wild-type C57BL/6 mice and in Skh:hr1 female mice treated with the ER antagonist ICI 182,780.

## 2. Results

### 2.1. Effect of 1,25(OH)2D on Photoprotection against CPDs in Female and Male Skh:hr1 Mice

**Figure 1:**
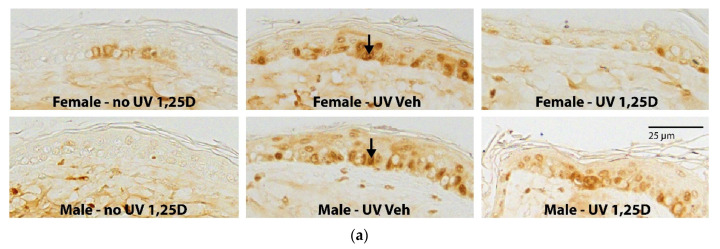

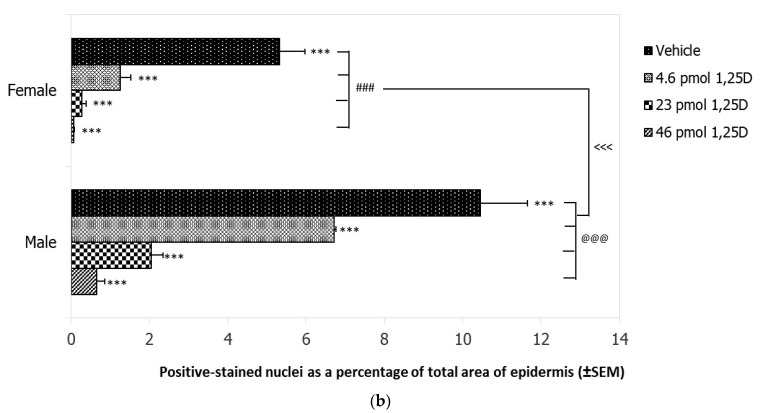
Protection against CPDs at 3 h post-SSUV by 1,25(OH)2D in female and male Skh:hr1 mice. Mice were exposed to 1 × 2.5 MED of SSUV followed immediately with topical vehicle or 1,25(OH)2D. (**a**) Skin sections, SSUV + vehicle or 1,25(OH)2D (23 pmol/cm^2^), stained immunohistochemically for CPD-positive nuclei. (**b**) Quantitation of the average percentage epidermal CPD-positive nuclear staining (*n* = 3 mice, 9 sections per group) following SSUV + vehicle or 1,25(OH)2D (4.6, 23 or 46 pmol/cm^2^). CPD staining in unirradiated skin was <0.1% positive nuclei. Results are representative of at least 2 experiments. *** significantly different from vehicle controls, *p* < 0.001. ###, @@@ significantly different among each concentration tested in the group, *p* < 0.001. <<< significantly different between females and males treated with vehicle, *p* < 0.001. <<< significantly different between females and males treated with the same concentration of 1,25(OH)2D, *p* < 0.001.

Figure 1a shows the immunohistochemical CPD-positive-stained nuclei in the epidermis at 3 h post-SSUV, with and without 23 pmol/cm^2^ of 1,25(OH)2D treatment. CPD immunostaining was below measurable levels in unirradiated skin, but was apparent in all irradiated samples and was significantly greater in vehicle-treated male mice (10.5 ± 1.2%) than in females (5.3 ± 0.7%; *p* < 0.001) (Figure 1b). Moreover, although the CPD staining was significantly reduced by topical 1,25(OH)2D in all groups (*p* < 0.001), the reduction was significantly more effective in female than in male mice (*p* < 0.001). Thus, the percentage protection against CPDs in female mice was 77 ± 0.4%, 95 ± 1.3%, and 99 ± 0.3% after topical treatment with 4.6, 23, and 46 pmol/cm^2^ 1,25(OH)2D, respectively, but in male mice this was reduced to 36 ± 0.1%, 80 ± 0.3%, and 94 ± 1.0% protection, respectively, after these doses of 1,25(OH)2D (Table 1). These results demonstrate that photoprotection against CPDs by 1,25(OH)2D was reduced in males compared to females treated with the same concentration of 1,25(OH)2D, with the protection in males being greatest at the higher doses of 1,25(OH)2D.

### 2.2. Effect of ER Blockade on 1,25(OH)2D Photoprotection against CPDs in Female

#### Skh:hr1 Mice

Skh:hr1 female mice express only ER-β but not ER-α in the epidermis, while Skh:hr1 male mice do not express any ER in the epidermis [38]. In order to test whether these male–female differences were dependent on the activity of the ER-β, we treated female Skh:hr1 mice, which do not express ER-α in the epidermis [38], for two weeks with the ER signaling inhibitor ICI 182,780). At 3 h post-SSUV, the relative CPD-positive nuclear staining was not significantly different in mice treated with vehicle alone or ICI 182,780 (100 ± 20% and 90 ± 5%, Table 1b). However, the reduction in CPD staining by 4.6, 23, and 46 pmol/cm^2^ 1,25(OH)2D was again highly significant (*p* < 0.001) and apparently dose-dependent, although this was not significant, at 81 ± 29%, 84 ± 19%, and 94 ± 23% protection, respectively. This protection against CPDs by 1,25(OH)2D was slightly, though not significantly, decreased by ICI 182,780 to 73 ± 19%, 74 ± 17%, and 74 ± 17%, respectively, but the role of ER-β could not be confirmed.

### 2.3. Effect of 1,25(OH)2D on SSUV-Induced CPDs in ER-β−/−Mice

SSUV induced a slightly but not significantly greater relative percentage of CPD-positive nuclear staining in ER-β−/−mice (125.7 ± 14.3%) than in wild-type C57BL/6 mice (100 ± 8.6%) (Table 1c). Topical 1,25(OH)2D at 23 pmol/cm^2^ significantly reduced the proportion of CPD staining in both ER-β−/− (68.6 ± 8.6%) and C57BL/6 mice (34.3 ± 2.9%), resulting in 44 ± 6% and 66 ± 8% protection, respectively (*p* < 0.05). This indicated that the photoprotection against CPDs was significantly reduced in ER-β−/−mice by 22 ± 3% (*p* < 0.05) compared to wild-type mice.

### 2.4. Effect of SSUV on Sunburn Cells in Female and Male Skh:hr1 Mice

The apoptotic keratinocytes (sunburn cell) numbers (in cells per linear millimeter as means ± SEM) were increased to the same extent in female and male Skh:hr1 mice from fewer than 2 sunburn cells/mm to 12.4 ±1.4 in female mice and 11.7 ± 4.3 in male mice. The reduction in sunburn cell numbers after topical 1,25(OH)2D was not significantly different between the sexes—7.1 ±1.3 in females and 4.5 ± 1.0 in males.

### 2.5. Effect of 1,25(OH)2D on SSUV-Induced Inflammatory Edema in Female and Male Skh:hr1 Mice

**Figure 2:**
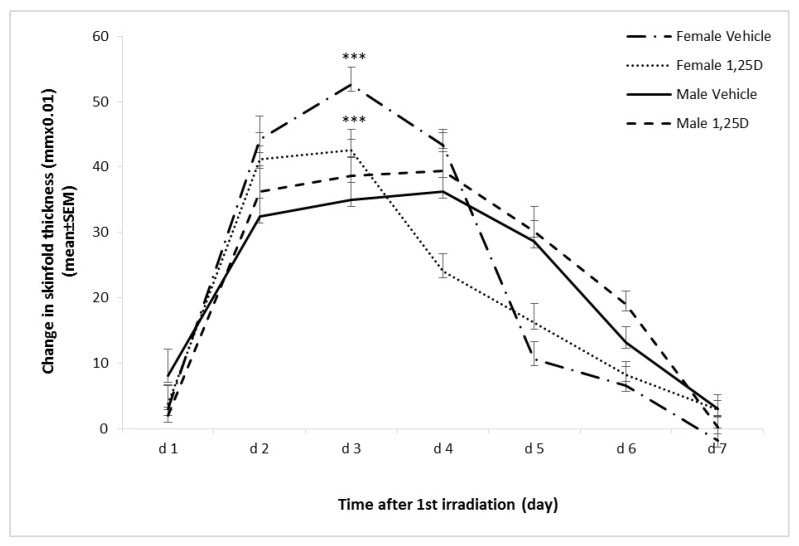
Protection against SSUV-induced inflammatory edema by 1,25(OH)2D in female and male Skh:hr1 mice. Mice (*n* = 5 per group) were exposed to 1 MED of SSUV for 3 consecutive days followed daily with topical vehicle, or 1,25(OH)2D (23 pmol/cm^2^). Average daily dorsal skinfold measurements ± SEM indicate edema compared to non-irradiated skinfold thickness. The data are representative of two independent experiments. *** matching symbols indicate statistically significant differences, *p* < 0.001.

Since erythema in the hairless mouse is difficult to observe, swelling of the skin, edema, is the method by which an inflammatory reaction can be quantitated [41]. In female mice, edema indicated by mid-dorsal skinfold thickness increased acutely, peaking at day 3 following the initial SSUV exposure, then decreasing rapidly by day 5 (Figure 2). In male mice, the skinfold thickness increased more gradually, was significantly less at day 3 (*p* < 0.001) than in females, reached a maximum on day 4, then decreased more slowly. Treatment with 23 pmol/cm^2^ 1,25(OH)2D significantly reduced the skinfold thickness in female mice from day 2 to day 4 compared to the vehicle-treated controls (*p* < 0.05), but the skinfold thickness did not respond to 1,25(OH)2D in male mice, suggesting that the anti-inflammatory activity of 1,25(OH)2D is ER-β-mediated.

### 2.6. Effect of 1,25(OH)2D Alone on Basal CHS in Male and Female Skh:hr1 Mice

**Figure 3:**
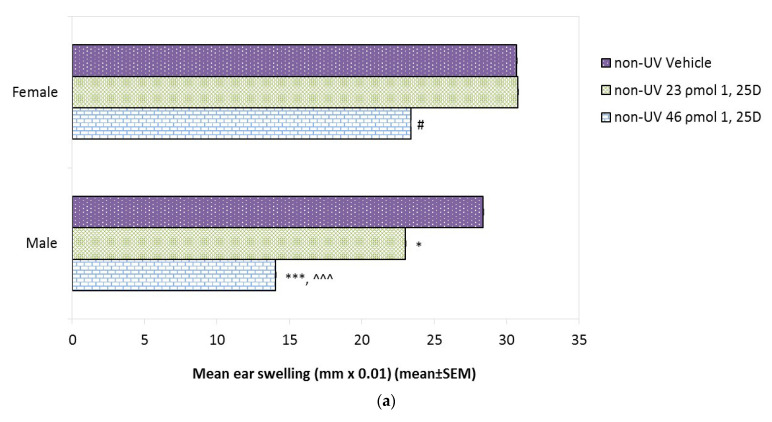

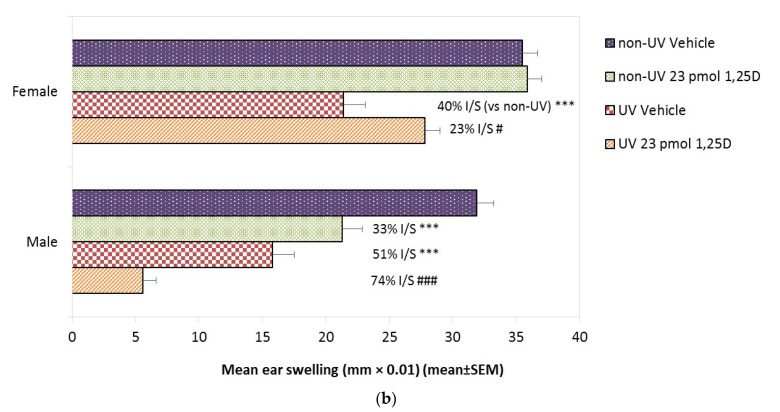
The effect of 1,25(OH)2D on the CHS reaction to oxazolone in female and male Skh:hr1 mice. (**a**) Effect of 1,25(OH)2D alone. Mice (*n* = 5 per group) were treated topically on the dorsal skin with vehicle or 1,25(OH)2D (23 or 46 pmol/cm^2^) for 3 consecutive days. They were sensitized a week later, challenged after a further week, and the CHS was assessed by ear swelling at 18 h. Data are pooled from four independent experiments. (SEM are too small to be visible on the graph). # significantly different from vehicle females *p* < 0.05. *** from vehicle males *p* < 0.001; * *p* < 0.05. ^^^ from 1,25(OH)2D (46 ρmol/cm^2^) females *p* < 0.001. (**b**) Effect of 1,25(OH)2D (23 pmol/cm^2^) post-SSUV. Mice were exposed to 1 MED of SSUV followed immediately with topical vehicle or 1,25(OH)2D (23 pmol/cm^2^) for 3 consecutive days, then sensitized one week later followed by challenge after a further week. Data are representative of two independent experiments (*n* = 5 mice per group). *** significantly different from non-UV controls *p* < 0.001. # from UV vehicle females *p* < 0.05. ### from UV vehicle males *p* < 0.001.

Since 1,25(OH)2D alone has been shown to be immunosuppressive in some mouse studies [42] and in humans [18], the effect on CHS of increasing doses of 1,25(OH)2D alone was assessed (Figure 3a). CHS was measured by the amount of ear swelling after oxazalone challenge, as described in Section 4.6, with greater ear swelling indicating a more robust immune response. In each of the figures below, the mean value for ear swelling for the five mice in each group is shown. The basal CHS reaction in vehicle-treated female and male mice was not significantly different (30.7 ± 0.05 and 28.4 ± 0.05 mm × 0.01, respectively). Topical 1,25(OH)2D was innocuous at 23 pmol/cm2 in females, although 46 pmol/cm^2^ resulted in a 25% suppression (*p* < 0.05). In contrast, in males there was a 14% suppression of CHS (*p* < 0.001) by 23 pmol/cm^2^ 1,25(OH)2D, which increased to 50% suppression by 46 pmol/cm^2^ 1,25(OH)2D (*p* < 0.001). Thus, the propensity for 1,25(OH)2D alone to suppress CHS was found to be dose-dependent and significantly more severe in males than in females.

### 2.7. Effect of 1,25(OH)2D on Photoimmune Suppression in Female and Male Skh:hr1 Mice

Whereas the basal CHS responses in unirradiated male and female mice again did not differ, exposure to 1 × 2.5 MED SSUV with vehicle was slightly although not significantly more immunosuppressive in male mice (51% suppression) than in females (40% suppression) (Figure 3b). In females, the application of 23 pmol/cm^2^ 1,25(OH)2D, though not immunosuppressive alone, significantly reduced the photoimmune suppression from 40% to 23% (*p* < 0.05). In males, already 33% immunosuppressed (*p* < 0.01) by the 1,25(OH)2D treatment alone, there was additional suppression after SSUV to 51% with vehicle and 74% suppression in the presence of 23 pmol/cm^2^ 1,25(OH)2D (*p* < 0.001 vs. SSUV-vehicle). Thus, topical 1,25(OH)2D protected against photoimmune suppression in female mice (*p* < 0.05) but exacerbated photoimmune suppression in male mice (Figure 3b), consistent with a role for estrogenic pathways in mediating photoimmune protection by 1,25(OH)2D.

### 2.8. Effect of ER-β Signalling Blockade on Photoimmune Protection by 1,25(OH)2D in Female Skh:hr1 Mice

**Figure 4:**
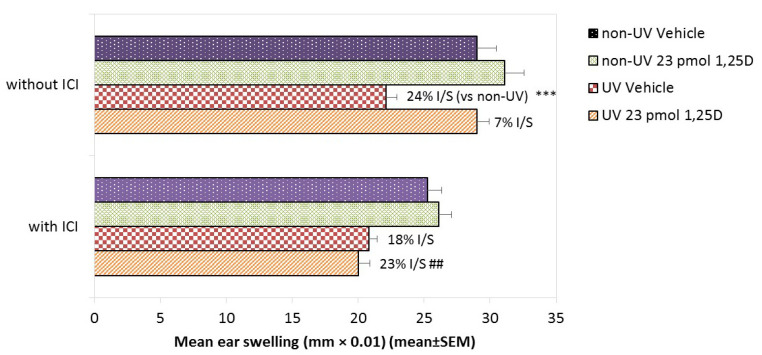
Effect of ICI 182,780 on protection by1,25(OH)2D against the SSUV-induced suppression of CHS in female Skh:hr1 mice. Mice (*n* = 5 per group) were pretreated topically with ICI 182,780 or vehicle for 2 weeks, then exposed to 1 MED of SSUV, followed immediately by topical vehicle or 1,25(OH)2D (23 pmol/cm^2^) on 3 consecutive days. ICI 182,780 or vehicle treatment was continued until sensitization with oxazolone one week later. Mice were challenged after a further week, and CHS assessed by ear swelling at 18. h. Data are representative of two independent experiments. *** significantly different from relevant non-UV control *p* < 0.001; ## *p* < 0.01.

The ER antagonist ICI 182,780 alone was slightly but not significantly immunosuppressive. SSUV significantly suppressed CHS similarly in both vehicle-treated (24% suppression) and ICI 182,780 pre-treated (18% suppression) mice (Figure 4). Topical 23 pmol/cm^2^ of 1,25(OH)2D again effectively reduced the photoimmune suppression to only 7 ± 0.6% (*p* < 0.001). However, ICI 182,780 abolished the protection by 1,25(OH)2D and CHS remained 23 ± 0.4% suppressed (*p* < 0.01). Thus, the pharmacological blockade of ER signaling prevented the photoimmune protective effect of topical 1,25(OH)2D, supporting the role of ER signaling in this process.

### 2.9. Effect of Genetic Deletion of ER-β on Photoimmune Protection by 1,25(OH)2D

**Figure 5:**
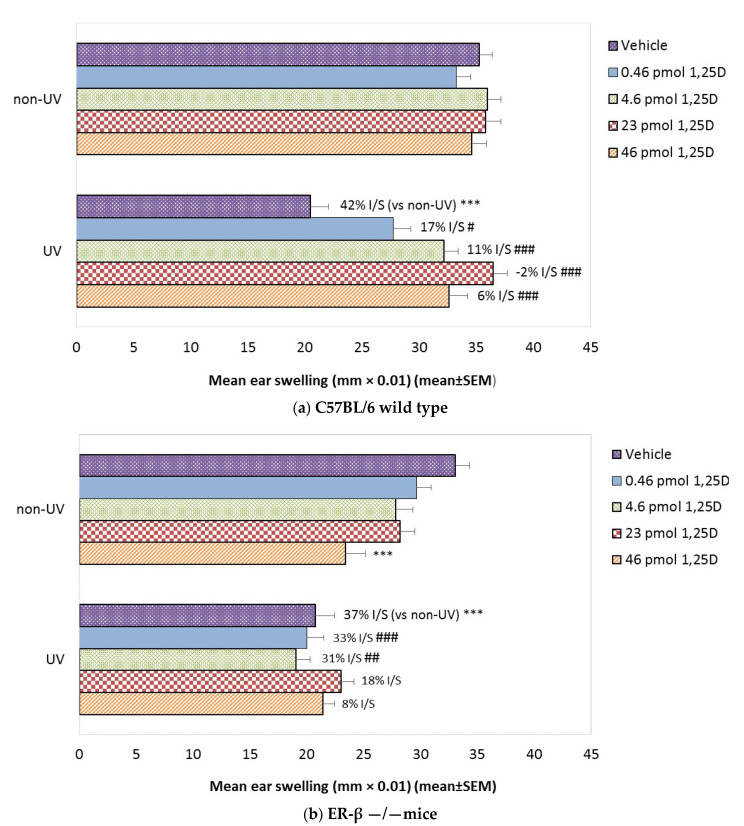
The effect of 1,25(OH)2D on the CHS reaction to oxazolone in female C57BL/6 wild-type and ER-β−/− mice. Mice (*n* = 5 per group) were exposed to 1 × 2.5 MED of SSUV (or not) and treated topically immediately with vehicle or 1,25(OH)2D (0.46, 4.6, 23, or 46 pmol/cm^2^. They were sensitized one week later, then challenged after a further week. Ear swelling was measured 18 h post-challenge. (**a**) Mean ear swelling in non-irradiated and SSUV-irradiated C57BL/6 wild-type mice. *** significantly different from relevant non-UV control *p* < 0.001. ### from UV vehicle *p* < 0.001; # *p* < 0.05. (**b**) Mean ear swelling in non-irradiated and SSUV-irradiated ER-β−/− mice. Data are representative of two independent experiments. *** significantly different from relative non-UV group *p* < 0.001; ### from UV vehicle *p* < 0.001; ## *p* < 0.01.

In C57BL/6 female mice (Figure 5a), increasing the doses of topical 1,25(OH)2D alone did not significantly alter the CHS response (unlike Skh:hr1 females). SSUV exposure suppressed CHS in vehicle-treated mice by 42%, but increasing the concentrations of 1,25(OH)2D, from 0.46, 4.6, 23.0, and 46 pmol/cm^2^ reduced the photoimmune suppression to 17%, 11%, −2%, and 6%, respectively. However, in ER-β−/− mice (Figure 5b) increasing the doses of 1,25(OH)2D alone appeared to suppress CHS, which was significant at the highest dose, 46 pmol/cm^2^ (*p* < 0.001). A similar degree of CHS suppression was observed after SSUV in vehicle-treated mice (37% suppression) as in the C57BL/6 mice, but the ER-β−/− mice treated with topical 1,25(OH)2D at 0.46 and 4.6 pmol/cm^2^ remained strongly immunosuppressed after SSUV exposure (33 and 31%), and only at 23 pmol/cm^2^ 1,25(OH)2D (18% suppression) and 46 pmol/cm^2^ (8% suppression) was there evidence of some photoimmune protection.

## 3. Discussion

These studies have probed the sex dependence of the responses of SSUV-irradiated mouse skin to topical 1,25(OH)2D applications. We have utilized the models of male versus female Skh:hr1 hairless mice, and, based on the published evidence of a photoprotective role for ER signaling specifically by ER-β, we have also examined the effects of the pharmacological blockade of the ER in female hairless mice and of the genetic deletion of the ER-β in female haired mice. A predilection for SSUV-induced DNA damage in the form of CPDs was observed in both male Skh:hr1 and female ER-β−/− mice that was dose-dependently reduced by topical 1,25(OH)2D. The higher doses for protection needed in male mice than in females were consistent with a dependence on estrogenic signaling, but significantly elevated CPD staining persisted in the epidermis of male and ER-β−/− mice. As the CPD, the major pre-mutagenic UV-induced DNA lesion is associated with immunosuppression and photocarcinogenesis [7,11,43]; our results are consistent with a sex bias reported by others in chronic UVB-induced photocarcinogenesis in Skh:hr1 mice. This revealed male mice to be at greater photocarcinogenic risk, accompanied by increased DNA damage measured as oxidative lesions [31]. In support, ovariectomized female mice were found to be equally susceptible to basal and squamous cell carcinomas as males [44].

Interestingly, although we observed increased rates of SSUV-induced DNA damage in male Skh:hr1 and in female ER-β−/− mice, followed by inhibited protection against CPD induction by topical 1,25(OH)2D, the treatment of Skh:hr1 females with the ER antagonist did not affect either the induction of CPDs by SSUV, nor provide significant evidence of inhibited protection by 1,25(OH)2D. In contrast, while the ER antagonist had no significant effect on the CHS response in Skh:hr1 females, it abrogated the immunoprotective effect of topical 1,25(OH)2D, similarly to the inhibited 1,25(OH)2D photoimmune protection in ER-β−/− mice. These contrary responses suggest that when the total ER expression is blocked, 1,25(OH)2D is unable to protect against CPDs, perhaps indicating that the major ER, ER-α, may contribute particularly in DNA protection by 1,25(OH)2D, but not necessarily in immune protection.

In addition, we observed sex-dependent differences in SSUV-induced inflammation in Skh:hr1 mice, with females developing a significantly greater but faster resolving edema in the irradiated skin than males, consistent with other reports [29]. The novel finding here was the inability of topical 1,25(OH)2D to ameliorate the progression of the inflammation in male mice, whereas topical 1,25(OH)2D caused a significant reduction in the edema in females. Other studies have shown that 1,25(OH)2D reduces the UV-induced pro-inflammatory cytokines interleukin (IL)-6 [45,46] and IL-12 [47,48], and thus may convert a pro-inflammatory to an anti-inflammatory state [49,50]. Cytokine responses to UV, however, are also affected by sex-related factors—for example, the enhanced UV induction of anti-inflammatory IL-10 or the reduced induction of pro-inflammatory IL-12 in ER-β−/− mice [38], suggesting this may modify photoprotection by 1,25(OH)2D against inflammation as well as immune function.

Our assessment of the effect of 1,25(OH)2D on SSUV-suppressed CHS reactions was complicated by the evidence that topical 1,25(OH)2D alone could suppress CHS and that this suppression was dose-dependently more severe in male than female Skh:hr1 mice and was also apparent in ER-β −/− mice, although not in female wild-type C57BL/6 mice. Others have reported that vitamin D compounds alone suppress T-helper type 1 (Th1) pro-inflammatory responses, modify cytokine expression patterns, induce the activation of Treg cells [42,51], inhibit antigen presenting cell maturation and function [18], and promote IL-10 release from cutaneous mast cells [52]. To explore how vitamin D might be both photoimmune protective and itself immunosuppressive, we tested a range of topical dosages of 1,25(OH)2D alone and demonstrated a basal dose-dependent immunosuppression in Skh:hr1 males at markedly lower concentrations than in females, and in ER-β−/− mice, but not in wild-type controls. We therefore conclude that immunosuppression by topical 1,25(OH)2D alone is both dose- and sex-dependent in mice, facts that could be confirmed in future studies by analyzing the inflammatory infiltrate for sex-dependent differences in SSUV-induced Treg cell populations using a broad range of 1,25(OH)2D doses. Since the VDR is present in various types of immune cells, immune responses are likely to be responsive to 1,25(OH)2D produced locally in the skin and to affect the CHS reaction.

We confirmed that CHS was more severely suppressed by SSUV in male than female Skh:hr1 mice [29], in agreement with the exacerbated suppression of CHS by UVB radiation reported by others in these male mice [53]; in female ER-β−/−mice associated with increased UVB-upregulated epidermal IL-10 [38]; and, in human subjects, males being more susceptible to photoimmune suppression than females [30].

Using a dose of 1,25(OH)2D that alone was innocuous in female mice but moderately immunosuppressive in Skh:hr1 males, we observed a significant attenuation of photoimmune suppression in female Skh:hr1 and C57BL/6 mice; in contrast, we observed the absence of this immune protection in female Skh:hr1 mice treated with the ER antagonist and in male Skh:hr1 and in ER-β−/−mice. In male Skh:hr1 mice, there was even a strong exacerbation by 1,25(OH)2D of the basally suppressed CHS reaction. These results are consistent with the ER-β playing a critical role in mediating photoprotection by topical 1,25(OH)2D.

Protection from UV-induced DNA damage by 1,25(OH)2D seems to require the presence of both the VDR and ERp57 (endoplasmic reticulum protein 57) [54]. The mechanisms involve an increase in p53 expression [22,24], the increased expression of NRf2 target genes in anti-oxidant pathways [22], and increased energy availability for DNA repair [14]. There is evidence of an association between the photoprotective pathways of 1,25(OH)2D and ER-β. Previous studies show that ER-β signaling upregulates the expression of VDR [40]. The natural ER ligand, 17β-estradiol, regulates the transcription and expression of the VDR in vivo [55,56] and in vitro [57] by binding to the ER-β and upregulating signal transduction through extracellular signal-regulated kinase (ERK) 1/2 and the activator protein 1 (AP-1) site in the VDR promoter [57]. In addition, the inhibition of estrogen synthesis and signaling by 1,25(OH)2D and its anti-inflammatory actions have been shown to be clinically crucial for inhibiting ER-positive breast cancer by reducing the levels of inflammatory prostaglandins and decreasing estradiol synthesis by aromatase selectively in breast cancer cells [58]. Furthermore, an inverse correlation of phytoestrogen consumption with colon tumor incidence was suggested to be a consequence of the enhanced colonic synthesis of 1,25(OH)2D [59]. Interestingly, a recent report shows that the serum 25-hydoxyvitamin D (25(OH)D) levels were significantly increased in vitamin D3-deficient female but not in deficient male mice after UV exposure, and were nevertheless immunosuppressed by erythemal UV irradiation [60]. These findings further emphasize the possible correlation between 1,25(OH)2D and ER-β in photoprotection. A similar immunological synergy between 1,25(OH)2D and the natural ligand 17β-estradiol has been observed in humans [61].

SSUV damage was clearly worse in male than in female mice, as appears to be the case for humans [30,32,33]. If vitamin D-derived compounds are ever used in topical sunscreens or after-sun lotions, to add biological enhancement to the physical barriers, the potentially higher concentrations needed for males may need to be considered.

## 4. Materials and Methods

### 4.1. Mice

These studies were approved by the Animal Ethics Committee of the University of Sydney (Protocol numbers K22/1-2011/3/5457—12 January 2011); 2015/794—1 May 2015). Age-matched groups of inbred male and female Skh:hr1 and female C57BL/6 mice were obtained from the University of Sydney Veterinary Science breeding colony at 9–12 weeks of age. A breeding nucleus of ER-β knockout mice on the C57BL/6 genetic background (homozygous ER-β −/− males mated with heterozygous ER-β +/− females) was obtained from the Jackson Laboratory (Bar Harbour, MA); the genotyping of the offspring provided the ER-β −/− female mice, also at 9–12 weeks of age. The mice were housed in groups of 3–5 in conventional wire-topped plastic boxes on compressed paper bedding (Fibrecycle Pty. Ltd., Mudgeeraba, QLD, Australia) at an ambient temperature of 25 °C under gold lighting (F40GO tubes, General Electric Co., Hobart, TAS, Australia) that does not emit UV, and fed stock rodent pellets (Gordons Specialty Stockfeeds, Yanderra, NSW, Australia) and tap water ad libitum. Because of their propensity to fight and bite, Skh:hr1 male mice remained housed with their original weaned male littermates throughout the experiments, and were not included if there were signs of skin damage. Haired mice were shaved with electric clippers (Oster, Milwaukee, WI, USA) at least 24 h before UV irradiation and were included in experiments while in the resting phase of hair growth (telogen ink skin).

### 4.2. UV Irradiation

The source of solar simulated UV radiation (SSUV; 290–400 nm) comprised of a bank of one central UVB (Philips TL40W 12/RS, Eindhoven, The Netherlands) and 6 UVA (Hitachi 40 W F40T 10/BL, Tokyo, Japan) fluorescent tubes, filtered through cellulose acetate sheeting (Grafix Plastics, Cleveland, OH, USA) as previously described [62,63]. Experiments were performed in a specially designed room with yellow fluorescent lights, which do not emit UV. Fluence was measured using a calibrated International Light IL1500 radiometer (Newburyport, MA, USA). The minimum erythemal dose (MED) of SSUV for female Skh:hr1 mice, measured by the mid-dorsal edema response at 24 h, was previously established as 1.33 kJ m^−2^ UVB and 23.7 kJ m^−2^ of UVA [62], and is approximately twice this for shaved pigmented haired mice. Groups of mice were exposed on the dorsum, unrestrained in their boxes with the wire tops removed, to either a single dose of 2.5 MED, or 3 repeated daily doses of 1 MED of SSUV as noted in the figure legends. C57BL/6 mice received 5 hairless mouse MEDs which is equivalent to 2.5 MEDs on Oster-clipped hairy skin, because of the residual fine stubble.

### 4.3. Immunohistochemical Detection of CPDs and Image Analysis

Mid-dorsal skin biopsies were collected at 3 h post-SSUV exposure, fixed in Histochoice (Amresco, Solon, OH, USA) for 6 h, processed in an automated ethanol-formalin system, and wax-embedded. Sections (4 μm) were stained with mouse monoclonal IgG1 λ anti-thymine dimer antibody H3 (Affitech, Oslo, Norway), together with the Animal Research Kit (Dako, Glostrup, Denmark), as previously described (Dixon et al., 2011). To control for specificity of the primary antibodies an isotype control at the same protein concentration was used, which resulted in no staining. Triplicate images from each of 3 mice per group were captured using Nikon Eclipse E800 microscope (Nikon Instruments Inc., Minato City, Japan) with Leica software (version 3.1.0 Leica Geosystems, Heerbrugg, St.Gallen, Switzerland) and analysed using Metamorph software (version 7.7, Molecular Devices, San Jose, CA, USA) to quantify the percentage of thymine dimer-positive nuclear staining in the selected area of epidermis, calculated by the formula: [1-(mean percent nuclear staining in SSUV with 1,25(OH)2D/mean percent nuclear staining in SSUV with vehicle) × 100] [64]. The percentage protection was calculated relative to the control SSUV treatment without 1,25(OH)2D. Thymine dimers, the commonest type of CPD, are correlated with other types of CPD [65]. We have previously shown that CPD results by immunohistochemistry and image analysis are very similar to those obtained using the T4N5 endonuclease detection of CPD, followed by Comet assay [19,21].

### 4.4. Sunburn Cells

Although sunburn cells are normally maximal around 24 h after SSUV exposure, they are readily detected at 3 h [66]. The use of skin sections at 3 h for sunburn cells as well as CPD, reduced the numbers of mice used in experiments, as mandated by the University of Sydney Animal Ethics Committee. Sections were rehydrated through graded alcohols and routine haematoxylin and eosin staining was carried out by Veterinary Pathology Diagnostic Service (University of Sydney). Sunburn cells were visualized as apoptotic keratinocytes with shrunken, elongated nuclei. The stained sections were examined under a Zeiss-Axioscan light microscope at 20× magnification, and the number of sunburn cells per linear millimeter of skin section was recorded as previously described [24].

### 4.5. The Inflammatory Edema Response

The inflammatory response to UV irradiation was recorded by measuring the mid-dorsal skin thickness of each mouse using a spring micrometer (Interrapid, Zurich, Switzerland). Control measurements were taken prior to irradiation and every 24 h following irradiation for 7 days or until levels returned to control levels. Haired mice were shaved prior to recording skin measurements and allowed to rest for several hours to prevent any inflammatory responses due to the shaving. The change in skin fold thickness was calculated by subtracting the pre-irradiation control values for each individual mouse.

### 4.6. Induction of Contact Hypersensitivity (CHS)

Groups of 5 mice were irradiated with either a single UV exposure or 3 consecutive daily UV exposures and treated topically, whereas control mice were treated but not irradiated. A week following UV exposure, all the mice were sensitized by applying 100 μL of 2% oxazolone (*w*/*v*) (Sigma-Aldrich, Castle Hill, NSW, Austalia) in 100% ethanol to the non-irradiated abdomen skin of each animal, as previously described [67]. Sensitization was repeated 24 h later to counter possible removal by grooming and to ensure replicable responses. A fortnight after UV exposure, ear thicknesses were measured using a spring micrometer before and after a challenge with 5 μL 2% oxazolone in 100% ethanol applied on each side of both ears (20 μL per mouse). Ear measurements were recorded at 16, 18, and 20 h post challenge to select the optimal experimental time point of maximum swelling in the vehicle unirradiated control mice. The percent immunosuppression was calculated at this time point from the differences between the matching unirradiated and SSUV-irradiated treatments, as previously described [24].

### 4.7. Topical Treatments

The 1α,25-dihydroxyvitamin D3 (1,25(OH)2D) stock (Cayman Chemical (Ann Arbor, MI, USA) in spectroscopic-grade ethanol (Merck KGaA, Darmstadt, Germany) was diluted to provide solutions in a vehicle of ethanol:propylene glycol (Sigma-Aldrich):water in the ratio of 2:1:1. Vehicle-treated mice were treated exactly the same as 1,25(OH)2D-treated mice, except for replacing treatment with ethanol. Immediately after irradiation, aliquots of 100 µL of 1,25(OH)2D or vehicle were applied to the dorsal skin. The complete ER antagonist ICI 182,780 (Tocris Bioscience, Ellisville, MO, USA) dissolved in acetone was applied to the dorsal skin in two doses of 5 nmol per week from 2 weeks before SSUV exposure, avoiding treatment immediately before irradiation to prevent possible screening effects, and for CHS experiments, continuing twice weekly until contact sensitization.

### 4.8. Statistical Analysis

The results are expressed as means ± SEM unless otherwise indicated. Coefficients of variation of the percent photoprotection were calculated using the coefficients of variation of each mean by the method of Colquhoun [68]. The statistical significance of the differences between treatment groups was obtained by one-way ANOVA followed by Tukey’s test, using Graphpad Instat 3.0 (GraphPad Software Inc., San Diego, CA, USA).

## 5. Conclusions

In conclusion, our study illustrates for the first time the sex bias in the protective role of topical 1,25(OH)2D against SSUV-induced inflammatory sunburn edema, DNA damage, and immunosuppression in independent mouse models. The inactivity of estrogenic pathways, specifically those regulated by ER-β signaling, significantly reduced the protective actions of topical 1,25(OH)2D against these critical cutaneous responses that might otherwise act to protect the skin from the initial steps towards skin cancer development and correlates with the relative insensitivity to 1,25(OH)2D photoprotection and greater skin cancer susceptibility known to prevail in the male sex.

## Figures and Tables

**Table 1:** Relative CPD induction at 3 h post-SSUV exposure. Quantitation of the effect of topical 1,25(OH)_2_D on CPD induction, obtained from the image analysis of the percent epidermal nuclear CPD-positive staining, relative to the vehicle (nil 1,25(OH)_2_D) in each normal ER functional mouse group. Triplicate images from each of 3 mice per group were analyzed and the percent protection by 1,25(OH)_2_D was calculated.

[1,25(OH)_2_D Dose] pmol/cm^2^	Protection %			Protection %
A	Female Skh:hr1 Mice	versus	Male Skh:hr1 Mice	
0 (vehicle)	100 ± 13.2		198.1± 22.6	
4.6	23.6± 5.1	77 **	126.4 ± 0.9	36 **
23	5.1 ± 1.9	95	39.7 ± 5.7	80
46	1.3 ± 0.2	99	12.3 ± 4.0	94
B	**Female Skh:hr1 Mice** **—ICI 182,780 versus**			+**ICI 182,780**
0 (vehicle)	100 ± 20.0		90.0 ± 5.0	
4.6	18.5 ± 0.4	81	24.0 ± 3.5	73
23	16.3 ± 1.5	84	23.5 ± 2.0	74
46	5.9 ± 0.8	94	23.5 ± 3.0	74
C	**Female C57BL/6 Mice**	**versus**	**Female ERβ−/−Mice**	
0 (vehicle)	100 ± 8.6		125.7 ± 14.3	
23	34.3 ± 2.9	66 *	68.6 ± 8.6	45 *

* significantly different, *p* < 0.05; ** significantly different, *p* < 0.001.

## Data Availability

Data are stored on the Research Data Store of the University of Sydney.
